# Evaluation of the ocular surface characteristics and *Demodex* infestation in paediatric and adult blepharokeratoconjunctivitis

**DOI:** 10.1186/s12886-019-1074-5

**Published:** 2019-03-07

**Authors:** Mengliang Wu, Xiaochun Wang, Jing Han, Tingting Shao, Yan Wang

**Affiliations:** 1 Department of Ophthalmology, Eye & ENT Hospital, Fudan University, 83 Fenyang Road, Xuhui District, Shanghai, 200031 China; 2 Key Laboratory of Myopia, Ministry of Health, Shanghai, China; 3 Ophthalmology Department of Zaozhuang Municipal Hospital, Zaozhuang, Shandong China

**Keywords:** Blepharokeratoconjunctivitis, Meibomian gland dysfunction, *Demodex*, Ocular surface

## Abstract

**Background:**

To evaluate the ocular surface characteristics and the infestation of *Demodex* in Chinese paediatric and adult blepharokeratoconjunctivitis (BKC).

**Methods:**

Fifty consecutive patients with BKC and 50 age- and sex-matched healthy subjects were enrolled. Lid margin characteristics and corneal disorders were evaluated under slit-lamp illumination. Four eyelashes were collected from each eye to examine *Demodex* infestation by light microscopy.

**Results:**

Corneal neovascularization (*P* = 0.001) and scarring (*P* = 0.040) were significantly worse in children than in adults with BKC, whereas meibum quality was worse in adults (*P* = 0.008). Diagnosis delay was longer in children with BKC than in adults (2.2 vs 1.2 years, *P* = 0.022). *Demodex* infestation was more frequent in subjects with BKC than in healthy subjects (56% vs 26%, *P* = 0.002). The lid margin inflammation and meibomian gland dysfunction were worse in *Demodex*-positive subjects than in *Demodex*-negative subjects with BKC.

**Conclusions:**

Children with BKC had severer corneal disorders compared with adult BKC patients, which may be caused by a long-delayed diagnosis. Ocular demodicosis was more common in subjects with BKC. Ocular *Demodex* infestation was associated with worse lid margin inflammation and meibomian gland dysfunction.

**Electronic supplementary material:**

The online version of this article (10.1186/s12886-019-1074-5) contains supplementary material, which is available to authorized users.

## Background

Blepharokeratoconjunctivitis (BKC) is a chronic and recurrent inflammatory disorder of the palpebral margin that is commonly associated with secondary conjunctival and corneal disorders [1, 2]. This disease is characterised by an inflamed lid margin (telangiectasia, thickening, and ulceration), meibomian gland dysfunction (MGD), and corneal disorders (punctate erosions, vascularisation, ulceration, and sometimes perforation) [3–5]. BKC affects the quality of life of patients, and due to the involvement of cornea, it may result in blindness [3, 5, 6]. However, BKC is often overlooked and misdiagnosed in the clinic [7, 8]. It was reported that BKC accounted for 12–25% of paediatric referrals for corneal diseases [9, 10]. But the delayed diagnosis is common, earlier studies showed the mean age at the onset of symptoms in children was 3.2–6.2 years and the mean age at diagnosis was 6.9–9.3 years [3, 5, 11]. So, the appropriate early diagnosis is very important in BKC. To understand the medical history and the ocular surface characteristics will be helpful for the early and correct diagnosis of BKC.

*Demodex* is a parasitic mite that lives in or near hair follicles and sebaceous glands. It was reported that *Demodex* was associated with chalazia, chronic blepharitis and MGD [12–15], as well as corneal manifestations such as corneal neovascularization, corneal infiltration, corneal opacity and scars, especially in patients with ocular rosacea [16]. However, there is scant research regarding its effect in BKC. Therefore, it will be valuable to examine the role of *Demodex* in the ocular surface changes of BKC.

In current study, we enrolled 50 consecutive BKC cases including children and adults to analyse the medical history and comprehensive ocular surface characteristics in BKC patients. We also compared the differences of clinical features between adult and pediatric BKC. Furthermore, we investigated the infestation of *Demodex* in BKC to evaluate the correlation between ocular *Demodex* and the ocular surface characteristics of patients with BKC.

## Methods

### Subjects

The study included 50 consecutive patients (50 eyes) who were first diagnosed with BKC at the Eye, Ear, Nose and Throat Hospital of Fudan University between April 2016 and March 2017. Patients were divided into two groups according to their age: patients aged ≤16 years were included in the paediatric group and patients aged > 16 years were included in the adult group. We also enrolled 50 age- and sex-matched healthy subjects (50 eyes) as a control group. All of the subjects were Chinese. The diagnostic criteria of BKC were as follows: (1) subjective symptoms, including tearing, photophobia, recurrent episodes of red eye, and (2) eyelid involvement, including anterior and/or posterior blepharitis (lid hyperemia, lid scaling or crusting, and lid margin hypertophy), chalazia and meibomitis; and (3) conjunctiva and cornea involvement, including conjunctivitis (conjunctival hyperemia, conjunctival chemosis, and conjunctival discharge), keratitis (superficial punctate keratopathy, corneal infiltration or ulceration), corneal vascularization, thinning, scarring or perforation. Subjects with other ocular surface diseases such as a history of atopy, vernal keratoconjunctivitis, or perennial allergic conjunctivitis, and a history of ocular surgery or systemic diseases that may affect ocular surface were excluded from the study. For the purpose of this study, we assessed the right eye or the affected eye in all subjects. Clinical data collection was approved by the Ethics Committee of Eye, Ear, Nose and Throat Hospital of Fudan University, in accordance with the Declaration of Helsinki.

### Medical history

The main symptoms at the time of the examination, the age at onset, treatment history, and history of chalazion were recorded in all subjects with BKC.

### Ocular examinations

All the subjects underwent slit-lamp examination, and ocular anterior segment photographs of the patients were taken. Ocular surface examinations included: (i) Tear breakup time (TBUT): The time before the first defect appeared on the stained tear film was recorded as the TBUT after the instillation of fluorescein. (ii) Schirmer’s I test (ST): Tear secretion test was measured by filter papers (Jingming New Technological Development Co. Ltd., Tianjin, China) placed in the lateral canthus for 5 min without topical anesthesia. (iii) Evaluation of corneal disorders: The corneal infiltration, ulceration, scarring and perforation was recorded. Corneal fluorescein staining of the upper, middle and lower parts of the cornea was assessed using a 4-point scale: no staining = 0; < 5 punctate stains = 1; 5 to < 10 punctate stains = 2; and 10 punctate stains or filamentous staining = 3. The total corneal staining score was determined as the summed score for all three parts, and ranged from 0 to 9 [17]. Corneal neovascularization was assessed on a range of 0–24 scale according to the number of quadrants affected, depth of involvement (superficial = anterior 1/3 of corneal thickness or deep = posterior 2/3) and location of vessels (peripheral = outer 1/3 of cornea, mid-peripheral = middle 1/3 and central = central 1/3). A score of 1 for each quadrant involved; a score of 1 was given for superficial vessels and 2 for deep vessels; and scores of 1, 2 and 3 for peripheral vessels, mid-peripheral vessels and central vessels respectively [18]. The lid margin was assessed to determine the presence of hyperaemia, telangiectasia and secretions (crusting and scaling on the eyelash). The meibomian gland was assessed in terms of meibum quality and meibum expressibility in accordance with the 2011 International Workshop on MGD [19]. Meibum quality was assessed in each of eight glands in the middle part of the lower eyelid, and scored on a 4-point scale: clear = 0; cloudy = 1; cloudy with particles = 2; and thickened, like toothpaste = 3. The scores for each eyelid were summed to generate a total score, which ranged from 0 to 24. Meibum expressibility was assessed in five glands in the middle parts of the lower eyelid with a 4-point scale according to the number of glands expressing meibum: all glands = 0; 3 or 4 glands = 1; 1 or 2 glands = 2; and no glands = 3. All examinations were completed by an experienced ophthalmologist at the same study site.

### Assessment of *Demodex* infestation

Two eyelashes were removed from each eyelid on the points of trisection (four lashes from one eye) by forceps under a slit-lamp microscope. The lashes from each eyelid were placed separately on a glass slide and mounted with a coverslip. To improve the ability of detecting and counting mites, each slide was stained with fluorescein, by wetting a fluorescein strip (Jingming New Technological Development Co. Ltd., Tianjin, China) with a drop of 0.9% NaCl solution and applying the strip to the edge of the coverslip until the lash was immersed [20]. *Demodex* mites were counted under a light microscope at magnifications of 50× and 100×. Each slide was assessed within 1 h of sampling by another researcher who didn’t know the patient information.

### Statistical analysis

All statistical analyses were performed using Stata 14.2 (StataCorp, College Station, TX, USA). Continuous variables are reported as the mean ± standard deviation. Categorical variables were recorded as the presence (yes) or absence (no), and reported as the number (percent) of subjects. Variables were compared between groups using Student’s t test, Wilcoxon’s rank-sum test, Pearson’s χ2 test, and Fisher’s exact test, as appropriate. In all analyses, *P* < 0.05 was considered statistically significant.

## Results

### Demographic characteristics and medical history

The paediatric BKC group included 18 patients (14 females and 4 males) with a mean age of 10.2 ± 3.8 years (range, 5–16 years). The adult BKC group included 32 patients (24 females and 8 males) with a mean age of 28.8 ± 7.1 years (range, 17–50 years). The paediatric control group included 17 children (12 females and 5 males) with a mean age of 9.3 ± 2.7 years (range, 6–16 years). The adult control group included 33 adults (22 females and 11 males) with a mean age of 32.2 ± 9.3 years (range, 17–52 years). The control and BKC groups were comparable in terms of the age and gender of subjects. Red eye was the main symptom in both groups, and was reported in 14/18 and 23/32 subjects, respectively. Recurrent chalazia affected 15/18 and 29/32 of subjects in the paediatric and adult BKC groups, respectively. The mean age of BKC related symptom onset was 7.9 ± 3.9 years in the paediatric group and 27.6 ± 7.5 years in the adult group; the mean diagnosis delay was 2.2 years and 1.2 years in the paediatric and adult BKC groups, respectively (*P* = 0.022). Before the diagnosis of BKC, many of the subjects had been diagnosed with viral keratitis or conjunctivitis; antiviral drugs had been prescribed to 29% of children and 28% of adults. The demographic characteristics and medical history of the paediatric and adult BKC groups are summarized in Table 1.

**Table 1:** The demographic characteristics and medical history of the paediatric and adult BKC groups

Characteristic	Paediatric BKC	Adult BKC	*P*-Value
Age (year, mean ± SD)	10.2 ± 3.8	28.8 ± 7.1	–
Gender (female/male)	3.5:1 (14/4)	3.0:1 (24/8)	1.000
Recurrent chalazia	67%(12/18)	75%(24/32)	0.529
Anti-virus treatment	29%(4/18)	28%(7/32)	1.000
Diagnosis delay (year, mean ± SD)	2.2 ± 1.8	1.2 ± 1.1	0.022^a^

*BKC* blepharokeratoconjunctivitis

^a^Statistically significant difference

### Clinical examination parameters

#### Adult BKC vs adult controls

TBUT was shorter in the adult BKC group than in the adult control group (*P* < 0.001), but the Schirmer I test was not significantly different among the two groups. The corneal staining score was significantly greater in adult BKC groups (*P* < 0.001). Regarding meibomian gland characteristics, the meibum quality and expressibility scores were significantly (both *P* < 0.001) worse in adult patients with BKC than in the respective control group.

#### Paediatric BKC vs paediatric controls

Comparison between children with BKC and those without found results similar to adult ones. Paediatric patients with BKC had a shorter TBUT and worse score of corneal staining, as well as poor meibum quality and meibum expressibility (all *P* < 0.001).

#### Paediatric BKC vs adult BKC

**Fig. 1:**
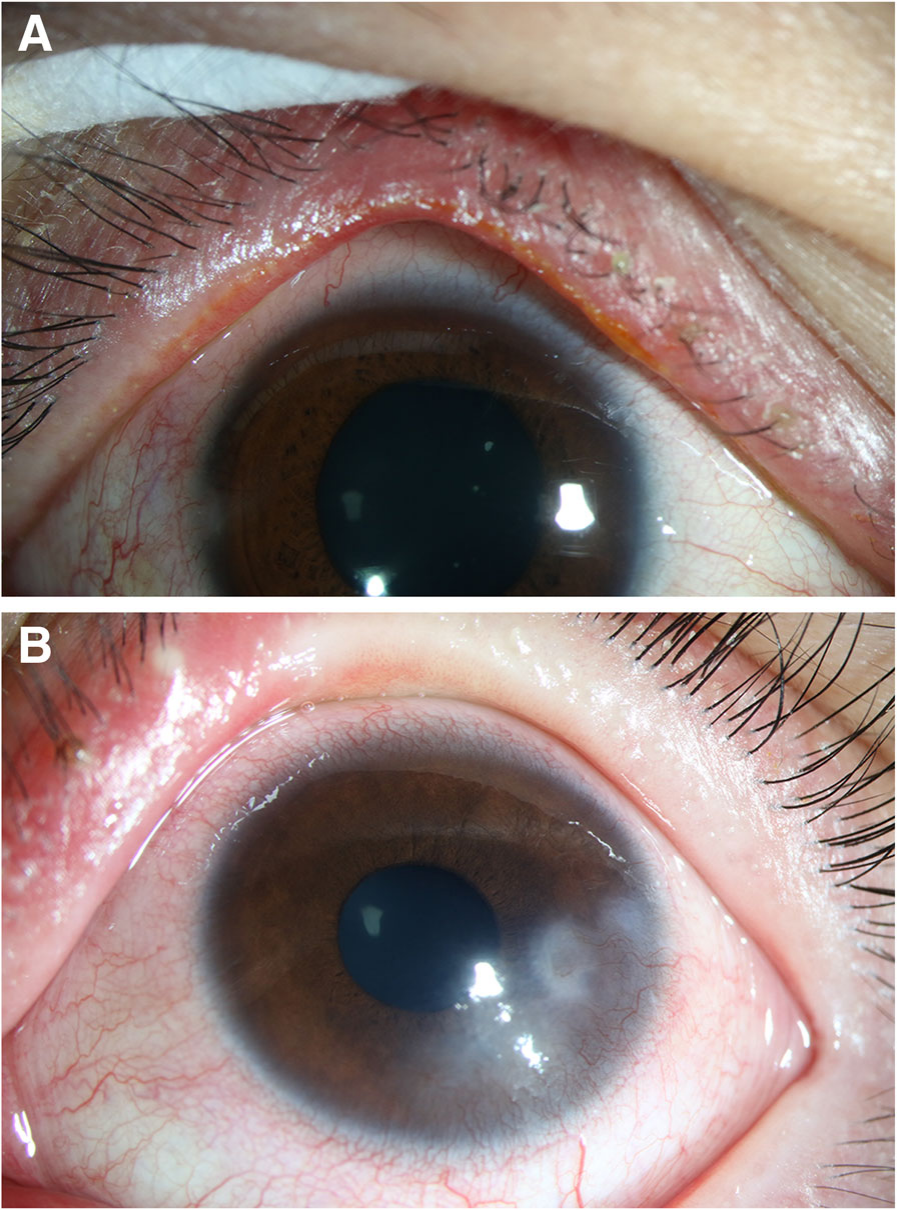
Representative slit-lamp photographs of paediatric and adult BKC. **a** Lid margin hyperaemia and secretion on the eyelashes in an adult. **b** Corneal opacity and scarring with lid margin hyperaemia in a child

Paediatric patients with BKC had a longer Schirmer’s I test result than the adult BKC group, but similar result was also found in child and adult controls. Regarding corneal disorders, corneal neovascularization (*P* = 0.001) and scarring (*P* = 0.040) were significantly worse in the paediatric BKC group than in the adult BKC group. Lid margin characteristics were not significantly different between the paediatric and adult BKC groups, except for the meibum quality score, which was significantly worse in the adult BKC group than in the paediatric BKC group (*P* = 0.008). Comparison of ocular surface characteristics between pediatric and adult BKC is summarized in Table 2 and representative slit-lamp photographs are shown in Fig. 1.

**Table 2:** Comparison of ocular surface characteristics between the paediatric and adult blepharokeratoconjunctivitis groups

	BKC		Control		P-value			
	Paediatric	Adult	Paediatric	Adult	(a)vs(c)	(b)vs(d)	(a)vs(b)	(c)vs(d)
	(a)	(b)	(c)	(d)				
TBUT (seconds)	3.7 ± 1.3	3.1 ± 0.9	8.2 ± 1.8	7.0 ± 2.3	< 0.001^a^	< 0.001^a^	0.097	0.310
ST (mm/5 min)	15.1 ± 8.8	11.0 ± 8.8	18.4 ± 8.8	13.2 ± 7.6	0.278	0.092	0.029^a^	0.037^a^
*Corneal disorder*								
Corneal staining	3.6 ± 1.8	2.8 ± 1.6	0.1 ± 0.2	0.1 ± 0.2	< 0.001^a^	< 0.001^a^	0.169	0.980
neovascularization	11.8 ± 4.3	4.3 ± 5.3	–	–	–	–	0.001^a^	–
infiltration	44%(8/18)	31%(10/32)	–	–	–	–	0.351	–
ulceration	6%(1/18)	13%(4/32)	–	–	–	–	0.642	–
scarring	61%(11/18)	31%(10/32)	–	–	–	–	0.040^a^	–
*Lid margin*								
hyperemia	50%(9/18)	69%(22/32)	–	–	–	–	0.190	–
telangiectasia	56%(10/18)	75%(24/32)	–	–	–	–	0.157	–
secretion	50%(9/18)	59%(19/32)	–	–	–	–	0.522	–
Meibum quality	4.1 ± 3.6	7.0 ± 3.0	0.3 ± 1.0	1.0 ± 1.6	< 0.001^a^	< 0.001^a^	0.008^a^	0.066
Meibum expressibility	1.3 ± 1.2	1.8 ± 1.2	0.0 ± 0.0	0.4 ± 0.7	< 0.001^a^	< 0.001^a^	0.175	0.019^a^

*BKC* blepharokeratoconjunctivitis, *TBUT* tear breakup time, *ST* Schirmer’s I test

^a^Statistically significant difference

### *Demodex* infestation and the clinical characteristics of BKC

*Demodex* infestation was more frequent in subjects with BKC than in healthy subjects (56% vs 26%, *P* = 0.002). We divided the 50 BKC patients into *Demodex*-positive and *Demodex*-negative groups, and the characteristics of both groups are shown in Table 3. The *Demodex*-positive group (28 patients) comprised 24 females and 4 males, and the mean age was 23.8 ± 10.9 years (range, 6–50 years). The *Demodex*-negative group (22 patients) comprised 14 females and 8 males, and the mean age was 19.9 ± 10.7 years (range, 5–38 years). TBUT and the corneal neovascularization score were not significantly different between *Demodex*-positive and *Demodex*-negative groups. However, the corneal fluorescein staining score was significantly greater in the *Demodex*-positive group than in the *Demodex*-negative group (*P* = 0.0015). Lid margin secretion (*P* = 0.013) was more common in the *Demodex*-positive group than in the *Demodex*-negative group. There were also significant differences in lid margin hyperemia and telangiectasia (*P* = 0.033 and 0.016, respectively), as well as meibum quality and expression scores (*P* = 0.019 and 0.028, respectively) between the *Demodex*-positive and *Demodex*-negative groups. Recurrent chalazion was more prevalent in the *Demodex*-positive group, although the difference was not statistically significant.

**Table 3:** Characteristics of *Demodex*-positive and *Demodex*-negative patients with blepharokeratoconjunctivitis

	*Demodex* -positive	*Demodex* -negative	*P* -Value
Age (year, mean ± SD)	23.8 ± 10.9	19.9 ± 10.7	0.215
Gender (female/male)	24:4	14:8	0.070
TBUT (seconds)	3.37 ± 1.24	3.33 ± 0.86	0.904
ST (mm/5 min)	12.4 ± 8.9	12.6 ± 9.2	0.911
*Cornea*			
Corneal staining	3.8 ± 1.6	2.2 ± 1.4	0.002 ^a^
Neovascularization	6.0 ± 6.0	7.7 ± 6.3	0.306
Infiltration	29%(8/28)	45%(10/22)	0.217
Ulceration	14%(4/28)	5%(1/22)	0.368
Scarring	43%(12/28)	41%(9/22)	0.890
*Lid margin*			
Secretion	71%(20/28)	36%(8/22)	0.013 ^a^
Hyperemia	75%(21/28)	45%(10/22)	0.033 ^a^
Telangiectasia	82%(23/28)	50%(11/22)	0.016 ^a^
Meibum quality	7.1 ± 3.0	4.5 ± 3.5	0.019 ^a^
Meibum expressibility	2.0 ± 1.1	1.2 ± 1.2	0.028 ^a^
Recurrent chalazia	79%(22/28)	64%(14/22)	0.243

*TBUT* tear breakup time, *ST* Schirmer’s I test

^a^Statistically significant difference

## Discussion

BKC is a chronic and recurrent inflammatory disease affecting the palpebral margin, and is accompanied with conjunctival and corneal lesions. BKC has a variety of manifestations, even neovascularization and scarring in severe cases, which may lead to impaired visual acuity. However, misdiagnosis or delayed diagnosis are common in BKC [3, 9]. In current study, we gave comprehensive examination on 50 consecutive patients with BKC to evaluate the characteristics of medical history and clinical symptoms. We also compared the difference of clinical parameters between adult and paediatric BKC patients. Furthermore, we investigated the potential role of ocular *Demodex* in this disease as well.

The gender distribution of BKC is inconsistent in different studies. A study of predominantly Caucasian patients found no difference in the proportion of males and females with BKC [9]. Another study of Asian children with BKC reported that 80% of the patients were female [21]. In current study, females accounted for a greater proportion of the enrolled BKC cohort than males among both children and adults. Considering these findings, the difference in gender distribution of BKC may be related to race. BKC seems to be prone to attack female in Asian. When we considered the mean age of patients, we found the mean age of paediatric and adult BKC in current study was 10.2 ± 3.8 and 28.8 ± 7.1 years respectively, which indicated BKC is more common in young female in Asian.

Delayed diagnosis in paediatric BKC attracted much more attention. In prior studies of paediatric BKC, the authors reported a delay between the onset and the correct diagnosis and treatment ranging from 1.3 to 2.4 years [3, 4, 9, 22]. In current study, we had a similar founding. We found the mean delay of diagnosis of BKC in children was about 2.2 years. And, even in adult, there is still a delay with 1.2 years, which suggests that the diagnosis of BKC is easy to be delayed in both children and adults. When we looked further the medical history of our subjects, we found viral keratitis and conjunctivitis were two common primary diagnoses in our patients. It was believed that herpes simplex virus keratitis was the most frequent misdiagnosis in patients with BKC [11]. In our study, 29% of children and 28% of adults received an anti-virus treatment before the diagnosis of BKC, indicating a previous misdiagnosis of virus keratitis. Because the long term and frequent use of anti-virus eye drops could destroy the ocular surface, the anti-virus treatment might aggravate the ocular surface damage of BKC, which indicates the importance of early correct diagnosis of BKC. The ignorance on the examination of eyelid margin might be the most important reason for the delayed diagnosis of BKC. On the other hand, the high incidence of recurrent chalazia in both paediatric and adult BKC groups indicated the recurrent chalazia could be a helpful clue for considering the diagnosis of BKC.

In current study, we compared the difference of clinical parameters between paediatric and adult BKC. We found the paediatric group had a higher corneal neovascularization score and worse corneal scarring than the adults. The severer corneal disorder in children BKC may be associated with the longer diagnostic delay and inappropriate primary treatment. It also indicated the possibility of different physiopathology between paediatric and adult BKC patients. It was reported that the effect on vision in children with BKC was more marked than one would expect in adults [3]. The authors believed that children may be more susceptible to corneal disorders from the inflammatory and immune response to periocular bacteria. However, the potentially different susceptibility and mechanism between paediatric and adult patients with BKC still needs further study. In spite of the less cooperativity in examination, lid margin examination still must be done in paediatric patients who complained chronic red eye, and especially when the patients had a history of recurrent chalazia. Many studies have reported that paediatric and adult BKC patients had worse meibomian gland function than people without BKC [3, 6, 23, 24]. Consistent with these earlier findings, the scores of meibum quality and meibum expression were significantly worse in both paediatric and adult BKC groups than that in the control groups in current study. However, except worse meibum quality in the adult BKC group, there was no significant difference in other lid margin parameters between the paediatric and adult BKC. Considering the worse corneal neovascularization and scarring in the paediatric BKC group, we think that the severity of corneal involvement may not be associated with the grade of lid margin inflammation. The association between the lid margin disorder and the corneal disorders still need further study to explore the potential physiopathology and specific treatments in paediatric BKC.

*Demodex* commonly congregates in eyelash follicles, sebaceous glands, including meibomian glands. It has been reported that *Demodex* plays an important role in blepharitis and ocular surface irritation [25–27] . However, there are still some researchers reported that the rate and extent of *Demodex* infestation was not significantly different between healthy people and patients with blepharitis [28]. In present study, 56% of patients with BKC were *Demodex* positive, with a statistically significant difference compared to 26% of the control group. The *Demodex*-positive patients had severer lid margin hyperaemia, telangiectasia, and secretion, and had worse meibomian gland function, which indicated that *Demodex* may be associated with the severity of lid margin disorders in BKC. The lid margin inflammation may be fortified by *Demodex* as a vector for bacteria [29] and via delayed hypersensitivity reactions [30]. Kheirkhah et al. [16] reported that patients with *Demodex* blepharitis presented with corneal staining and neovascularization, both of which were significantly reduced after treating the lid with tea tree oil to kill ocular *Demodex* [31]. However, current study only showed worse corneal staining in the *Demodex*-positive patients with BKC, there was no obvious difference in other corneal disorder parameters between the *Demodex*-positive and *Demodex*-negative BKC patients. Based on the findings, we speculated that the increased infestation of *Demodex* in BKC might be secondary to the lid margin disorder and conversely it could aggravate the lid margin and corneal disorders. So, the infestation of *Demodex* should be considered when we diagnose and treat the BKC.

Our study had several limitations. Although the lash sampling and microscopic examination was commonly used to identify the mites in the lashes, especially easy performed on children, it may miss the *Demodex* accumulated in the follicles of eyelashes. In vivo confocal microscopy (IVCM) may provide a more complete examination of the follicles [32]. Another consideration is that the reliability of meibomian glands examinations in paediatric patients which should be interpreted with caution. Moreover, although the simple slit-lamp analysis could partially elucidate the lid margin and corneal physiopathology of BKC, for further understanding the physiopathology mechanism of BKC, the analysis of tear of impression cytology will be necessary in the future study.

## Conclusions

In conclusion, we gave a comprehensive examination on the medical history and clinical parameters of 50 consecutive paediatric and adult BKC patients. We found that children with BKC had severer corneal disorders and longer delayed diagnosis compared with adult BKC patients. Ocular demodicosis was more common in subjects with BKC. Ocular *Demodex* infestation was associated with worse lid margin inflammation and meibomian gland dysfunction in BKC patients. Based on these findings, we thought a future study with a larger number of patients will be necessary to reveal the association between the lid margin disorders and the corneal disorders, and to identify the specific role of *Demodex* on the clinical parameters in BKC. Further study with the support of impression cytology and IVCM is also necessary to investigate the possibly different physiopathology mechanism between paediatric and adult BKC.

## Additional file

Additional file 1: Raw data for BKC study. Clinical data of ocular surface characteristics in controls and BKC patients. (XLSX 17 kb)

## Abbreviations

BKC: Blepharokeratoconjunctivitis
IVCM: In vivo confocal microscopy
MGD: Meibomian gland dysfunction
ST: Schirmer’s I test
TBUT: Tear breakup time
